# Endogenous hydrogen sulphide attenuates NLRP3 inflammasome-mediated neuroinflammation by suppressing the P2X7 receptor after intracerebral haemorrhage in rats

**DOI:** 10.1186/s12974-017-0940-4

**Published:** 2017-08-18

**Authors:** Hengli Zhao, Pengyu Pan, Yang Yang, Hongfei Ge, Weixiang Chen, Jie Qu, Jiantao Shi, Gaoyu Cui, Xin Liu, Hua Feng, Yujie Chen

**Affiliations:** Department of Neurosurgery, Southwest Hospital, Third Military Medical University, 29 Gaotanyan Street, Shapingba District, Chongqing, 400038 China

**Keywords:** Hydrogen sulfide, Intracerebral haemorrhage, NLRP3 inflammasome, P2X7 receptor, Microglia

## Abstract

**Background:**

Emerging studies have demonstrated the important physiological and pathophysiological roles of hydrogen sulphide (H_2_S) as a gasotransmitter for NOD-like receptor family pyrin domain-containing 3 (NLRP3) inflammasome-associated neuroinflammation in the central nervous system. However, the effects of H_2_S on neuroinflammation after intracerebral haemorrhage (ICH), especially on the NLRP3 inflammasome, remain unknown.

**Methods:**

We employed a Sprague–Dawley rat of collagenase-induced ICH in the present study. The time course of H_2_S content and the spatial expression of cystathionine-β-synthase (CBS) after ICH, the effects of endogenous and exogenous H_2_S after ICH, the effects of endogenous and exogenous H_2_S on NLRP3 inflammasome activation under P2X7 receptor (P2X7R) overexpression after ICH, and the involvement of the P2X7R in the mechanism by which microglia-derived H_2_S prevented NLRP3 inflammasome activation were investigated.

**Results:**

We found ICH induced significant downregulation of endogenous H_2_S production in the brain, which may be the result of decreasing in CBS, the predominant cerebral H_2_S-generating enzyme. Administration of *S*-adenosyl-l-methionine (SAM), a CBS-specific agonist, or sodium hydrosulfide (NaHS), a classical exogenous H_2_S donor, not only restored brain and plasma H_2_S content but also attenuated brain oedema, microglial accumulation and neurological deficits at 1 day post-ICH by inhibiting the P2X7R/NLRP3 inflammasome cascade. Endogenous H_2_S production, which was derived mainly by microglia and above treatments, was verified by adenovirus-overexpressed P2X7R and in vitro primary microglia studies.

**Conclusions:**

These results indicated endogenous H_2_S synthesis was impaired after ICH, which plays a pivotal role in the P2X7R/NLRP3 inflammasome-associated neuroinflammatory response in the pathogenesis of secondary brain injury. Maintaining appropriate H_2_S concentrations in the central nervous system may represent a potential therapeutic strategy for managing post-ICH secondary brain injury and associated neurological deficits.

## Background

Intracerebral haemorrhage (ICH) is a devastating stroke subtype with high mortality and morbidity rates. Numerous forms of evidence from preclinical and clinical studies suggest that inflammatory mechanisms are involved in the pathophysiologic progression of ICH-induced secondary brain injury [1, 2]. Our previous study demonstrated that NOD-like receptor (NLR) family pyrin domain-containing 3 (NLRP3) inflammasome activation contributes greatly to neuroinflammation development after ICH [3]. Despite the performance of several recent studies, in which the NLRP3 inflammasome was manipulated in experimental ICH animals [4–6], the fundamental mechanisms underlying NLRP3 inflammasome-mediated neuroinflammation are still unclear.

Hydrogen sulphide (H_2_S) is a colourless gas with a special rotten egg odour. H_2_S has been considered a toxic and potentially lethal gas for centuries, but emerging studies have demonstrated its important physiological and pathophysiological roles as a gasotransmitter in the central nervous system and in other tissues [7]. Relatively high H_2_S concentrations (50–160 mol/L) have been observed in the brain tissues of many species, including humans [8]. H_2_S plays a significant neuroprotective role in central nervous system diseases, including Alzheimer’s disease, Parkinson’s disease, traumatic brain injury, subarachnoid haemorrhage and ischaemic stroke, as a result of its anti-inflammatory and anti-oxidative effects [7, 9, 10]. However, the effects of H_2_S on neuroinflammation after ICH, especially its effects on the NLRP3 inflammasome, remain unknown.

The purinergic P2X7 receptor (P2X7R) is an ATP-gated, non-selective cation channel that belongs to the ionotropic P2X receptor family. The P2X7R is mainly expressed on immune cells, can be activated by high concentrations of extracellular ATP released by any type of cell injury and can regulate innate immune and inflammatory responses [11]. It was reported that the P2X7R directly interacted with NLRP3 inflammasome scaffold protein and was responsible for NLRP3 recruitment and activation [12]. In the case of ICH, P2X7R levels and NLRP3 inflammasome component levels were both significantly elevated and reached their peak at 1 day after ICH onset [13].

Based on these findings and the results of our previous study, we postulate that endogenous H_2_S attenuates NLRP3 inflammasome-mediated inflammatory injury and may be involved in the P2X7R signalling pathway. To explore this hypothesis, we sought to investigate the anti-inflammatory effects of microglia-derived endogenous H_2_S in the setting of ICH, both in vivo and in vitro, as well as the involvement of P2X7R signalling in this process.

## Methods

### Experimental animals

A total of 299 Sprague–Dawley (SD) male rats weighing 280–320 g and 40 1-day-old post-natal Sprague–Dawley male rats were provided by the Experimental Animal Centre of the Third Military Medical University (Chongqing, China). All experimental procedures and animal care procedures were approved by the Ethics Committee of Southwest Hospital, performed in accordance with the guidelines by the National Institutes of Health Guide for the Care and Use of Laboratory Animals, and reported following the ARRIVE guidelines (Animal Research: Reporting in Vivo Experiments, https://www.nc3rs.org.uk/arrive-guidelines). The rats were housed in a temperature- and humidity-controlled environment, were provided food and water ad libitum, were maintained under a 12-h light/dark cycle and were acclimatized for more than 1 week before undergoing surgery.

### Experimental protocol

In the present study, the following four separate experiments were conducted:

#### Experiment 1

To investigate the time course of H_2_S content and the spatial expression of cystathionine-β-synthase (CBS) after ICH, we divided 50 rats into eight groups (sham and 3, 6, 12 h, 1 2, 3 and 7 days after ICH, *n* = 6). Brain striatum H_2_S content and plasma and protein CBS expression were assessed by methylene blue assay and western blotting, respectively. Tissue samples for immunofluorescence were collected from two additional rats 1 day after ICH induction.

#### Experiment 2

To evaluate the effects of endogenous and exogenous H_2_S after ICH, we randomized 149 rats into the following four groups: sham, vehicle (ICH + saline, intraperitoneal injection), *S*-adenosyl-l-methionine (SAM), and sodium hydrosulfide (NaHS). The sham group did not undergo the measurements of H_2_S content and haemorrhagic volume and the detection of leukocyte infiltration and microglia accumulation (*n* = 4). Methylene blue testing (*n* = 6), haemoglobin assay (*n* = 5), western blotting (*n* = 6), quantitative polymerase chain reaction (qPCR, *n* = 6), Fluoro-Jade C staining (*n* = 5), immunofluorescence (*n* = 4) and terminal deoxynucleotidyl transferase dUTP nick end-labelling (TUNEL) staining (*n* = 4) were carried out 1 day after ICH induction, and Modified Neurological Severity Scores (mNSSs, *n* = 6) and brain water content (*n* = 6) were assessed at both 1 and 3 days after ICH.

#### Experiment 3

To evaluate the effects of endogenous and exogenous H_2_S on NLRP3 inflammasome activation under P2X7R overexpression after ICH, 100 rats were randomized into the following five groups: vehicle (ICH + saline, intracerebroventricular injection), adenovirus GFP (Ad-GFP) (as a control to adenovirus P2X7R (Ad-P2X7R)), Ad-P2X7R, Ad-P2X7R + SAM, and Ad-P2X7R + NaHS. For the 3-day study, mNSSs (*n* = 5 for the 1-day or 3-day time point) and brain water content (*n* = 5 for the 1-day or 3-day time point) were assessed. qPCR (*n* = 5) and western blotting (*n* = 5) were performed at 1 day after ICH.

#### Experiment 4

To validate the involvement of the P2X7R in the mechanism by which microglia-derived H_2_S prevented NLRP3 inflammasome activation, we harvested rat primary microglial cells from 40 1-day-old post-natal rats, cultured the cells and randomized them into the following four groups: control, LPS + ATP, LPS + ATP + SAM and LPS + ATP + NaHS. qPCR (*n* = 3) and western blotting (*n* = 3) were performed, and the effects of different SAM and NaHS concentrations on cell viability (*n* = 3) were measured by Cell Counting Kit-8 (CCK-8) under lipopolysaccharide (LPS) and ATP stimulation.

### Drug and adenovirus administration

*S*-adenosyl-l-methionine, a CBS-specific agonist (SAM, Sigma-Aldrich, St. Louis, MO, USA), and sodium hydrosulfide, a classical exogenous H_2_S donor (NaHS, Aladdin, Shanghai, China), were diluted to concentrations of 100 mg/kg [14, 15] and 5.6 mg/kg [7], respectively, in vehicle (saline) solution. The rats were treated intraperitoneally with SAM, NaHS or vehicle at 0.5 h after ICH. For the 72-h study, SAM, NaHS and vehicle were administered three times at 0.5, 24, and 48 h after ICH.

For in vivo adenovirus administration, the following two types of recombinant adenoviruses were used for gene transfer: (1) a replication-deficient human adenovirus containing the rat P2X7R (adenovirus P2X7R, Ad-P2X7R), which was used to upregulate P2X7R expression, and (2) an adenovirus containing human GFP (Ad-GFP), which served as a control for Ad-P2X7R. Ad-P2X7R (6 × 10^10^ pfu/mL) and Ad-GFP (2 × 10^10^ pfu/mL) were produced by Genechem in Shanghai, China. Both adenoviruses were stored at − 80 °C until use and were diluted to 1.3 × 10^10^ pfu/mL in an enhanced transfection solution (Genechem, Shanghai, China) before intracerebroventricular injection. A total volume of 10 μL was used for each adenovirus administration to the animals.

### Intracerebroventricular injection and ICH model establishment

The adenoviruses were injected into the left lateral ventricle 6 days before ICH, according to the previous procedure [16]. The rats were anaesthetised with intraperitoneal injections of sodium pentobarbital (100 mg/kg, Sigma-Aldrich, St. Louis, MO, USA) and then fixed onto a stereotaxic head apparatus. A 26-gauge needle in a 10-μL Hamilton syringe (Microliter 701; Hamilton Company, Reno, NV, USA) was inserted into the left lateral ventricle through a cranial burr hole at the following coordinates relative to the bregma: 1.5 mm posterior, 3.0 mm lateral and 5.5 mm below the horizontal plane of the bregma. Then, the abovementioned formulated solution was slowly injected by a microinfusion pump (Harvard Apparatus, Holliston, MA, USA) at a rate of 0.5 μL/min. The needle was kept in place for 10 min after injection before being slowly withdrawn, and then, the head of the rat was tilted nose-down at a 30° angle for 30 min after suture placement. The animals in the sham and vehicle groups were injected with equal volumes of saline into their left lateral ventricle, and then, their incisions were closed with sutures.

The experimental ICH model was established as described in our previous study [17]. Briefly, the rats were anaesthetised and positioned prone in a stereotactic head frame (Kopf Instruments, Tujunga, CA, USA). A scalp incision was made along the midline, and a burr hole (1 mm) was drilled on the right side of the skull at 3 mm lateral to the bregma and 5 mm ventral to the cortical surface. One microliter (0.2 μL/min) of saline containing 0.2 units of bacterial collagenase (type VII; Sigma-Aldrich, St. Louis, MO, USA) was injected stereotaxically into the striatum (6 mm ventral from the skull surface) using a Nanomite Syringe Pump (Harvard Apparatus, Holliston, MA, USA). After injection, the syringe was left in place for 5 min. The needle was slowly removed over an additional 5 min to prevent backflow. The hole was sealed with bone wax, and the wound was sutured closed. In the sham group, the rats were subjected to only the needle insertion procedure described above. The rats were allowed to recover in separate cages, with free access to food and water.

### Primary microglial cultures and drug treatment

Primary cultured rat microglial cells were prepared as described in our previous study [18], with minor modifications. Briefly, the meninges, choroidal plexus, brainstem and cerebellum were carefully separated from the cerebral hemispheres of 1-day-old post-natal Sprague–Dawley rats. Then, the cortices of the cerebral hemispheres were dissected and digested with 0.25% trypsin. The fragments were washed twice with D-Hank’s solution, and the resuspended cells were then collected and seeded in uncoated culture flasks containing the indicated medium (DMEM/F12 supplemented with 10% foetal bovine serum, 1 × 10^5^ U/L penicillin, 1 × 10^5^ U/L streptomycin sulphate, pH 7.2) at a concentration of 1 × 10^6^ cells/mL. The cells were cultured at 37 °C in humidified 5% CO_2_/95% air, and the medium was changed every 4–5 days. Upon reaching confluence (14 days), the microglial cells were separated from the underlying astrocytic monolayer via gentle shaking of the flasks overnight, a separation made possible by the differential adhesive properties of the two cell lines. The floating microglia were subsequently collected and plated on sterile culture dishes at a density of 1 × 10^5^ cells/cm^2^. After 24 h of culture, the microglial cells were ready for use. The cells were verified via immunohistochemical staining with microglia-specific Iba1 antibodies.

To induce inflammasome activation, we plated 1 × 10^5^ cells in a 6-well plate overnight. The medium was changed to serum-free medium the following morning, and then, the cells were treated with 1 μg/mL lipopolysaccharide (LPS, Sigma, St. Louis, MO, USA) with SAM or NaHS for 12 h. We administered dimethylsulfoxide (DMSO, Sigma, St. Louis, MO, USA)-only treatment as a control. Then, the microglial cells were exposed to 5 mM adenosine triphosphate (ATP) for 1 h. The cell extracts and precipitated supernatants were analysed by immunoblotting.

### Neurobehavioural evaluation

Neurobehavioural function was assessed with the Modified Neurological Severity Scores (mNSSs) at 1 and 3 days after ICH by an investigator who was blinded to the experimental groups. The mNSS (normal score, 0; maximal deficit score, 18) is determined by summing the scores of motor, sensory, reflex and balance assessments [19], with higher scores signifying more severe neurological deficits.

### Brain water content

Brain water content was evaluated for the brain edema as previously reported [16]. Briefly, the brain specimens were quickly divided into the ipsilateral cortex (Ipsi-CX), ipsilateral basal ganglia (Ipsi-BG), contralateral cortex (Cont-CX) and contralateral basal ganglia (Cont-BG). Brain water content was calculated as (wet weight − dry weight)/wet weight × 100%.

### Methylene blue assay

Methylene blue assay was performed for the H_2_S concentration. The rats were deeply anaesthetised, and their brains were removed post-operatively. The ipsilateral striatum was dissected out on an ice-cold operating table and immediately frozen at −80 °C until assayed. The tissues from the striatum of the injured hemisphere were collected for determination of their H_2_S concentrations by the spectro-luminosity method. The brain tissues were homogenized in ice-cold 50 mmol/L potassium phosphate buffer, pH 8.0 (12% wt/vol), with a Polytron homogenizer. The tissue homogenates were centrifuged (47,000*g*, 10 min, 4 °C), and the cell supernatants were collected to evaluate H_2_S levels. Blood samples were collected from the abdominal aorta and were immediately centrifuged to obtain plasma to test H_2_S levels. The cell supernatants or plasma samples (75 μL) were mixed with 0.25 mL of Zn acetate (1%) and 0.45 mL of water for 10 min at room temperature. Trichloroacetic acid (TCA; 10%, 0.25 mL) was then added to the mixture, which was centrifuged under the indicated conditions (14,000*g*, 10 min, 4 °C). The clear supernatant was collected and mixed with *N*,*N*-dimethyl-*p*-phenylenediamine sulphate (20 mmol/L; 133 μL) in 7.2 mol/L hydrogen chloride (HCl) and ferric trichloride (FeCl_3_; 30 mmol/L, 133 μL) in 1.2 mol/L HCl. After the resulting solution had incubated for 20 min at room temperature, we measured its absorbance at 670 nm with a spectrophotometer. A calibration curve of absorbance versus sulphide concentrations was constructed using defined concentrations of NaHS solution. When NaHS is dissolved in water, HS− is released and forms H_2_S with H+. The H_2_S concentration was taken as 30% of the NaHS concentration in each calculation.

### Haemoglobin assay

The rats were perfused transcranially with phosphate-buffered saline under anaesthesia, and their ipsilateral hemispheres were collected for the following haemoglobin assay to indicate the hematoma volume, as previously reported [20]. Briefly, the rat ipsilateral hemispheres were homogenized in 3 mL of distilled water for 60 s. Then, the homogenized specimens were centrifuged at 13,000*g* for 30 min, after which 400 μL of Drabkin’s reagent (Sigma-Aldrich, St Louis, MO, USA) was added to 100 μL of supernatant and stored at room temperature for 15 min. The haemoglobin absorbance was measured at 540 nm by a spectrophotometer and quantified using a standard curve. The results are presented as microliters of blood to represent the haemoglobin content in the ipsilateral hemispheres.

### Fluoro-Jade C staining

Perihaematomal neuronal degeneration was examined by Fluoro-Jade C staining (Millipore, Temecula, CA, USA), as described previously [17]. The brain tissue sections were rinsed in basic alcohol for 5 min before being rinsed for 2 min in 70% alcohol. Distilled water was used to remove the alcohol, after which the sections were incubated in 0.06% potassium permanganate (KMnO_4_) for 10 min, stained with 0.0001% Fluoro-Jade C in 0.1% acetic for 10 min and rinsed in distilled water for 5 min. After being triple-rinsed with distilled water, the sections were air-dried for 10 min, cleared in xylene and then covered with slips. Four perihaematomal images in each section were captured by a Zeiss microscope (Zeiss AxioCam, Germany), and Fluoro-Jade C-positive neurons were counted by ImageJ (National Institutes of Health, Bethesda, MD, USA).

### CCK-8 assay

Primary microglial cells (1 × 10^5^ cells/mL) were seeded into 96-well plates (100 μL/well) and grouped according to the different concentrations of SAM (0, 10, 100, 20, 400 μmol/L) and NaHS (0, 10, 100, 20, 400, 600 μmol/L) with which they were treated under LPS and ATP stimulation. Cell viability was evaluated by CCK-8 assay (Dojindo Laboratories, Kumamoto, Japan). At the end of each time point, the medium in the 96-well culture plates was changed to DMEM/F12 to avoid background interference, and then, CCK-8 (10 μL) was added to each well. Absorbance was measured at 450 nm using a spectrophotometer, and 620 nm was used as a reference wavelength.

### qPCR

Total RNA was extracted from perihaematomal brain tissue specimens or primary microglial cells using TRIzol reagent (Invitrogen, Camarillo, CA, USA). Isolated RNA was reverse-transcribed into complementary DNA (cDNA) using a cDNA synthesis kit (Vazyme, Jiangsu, China) in accordance with the manufacturer’s protocols. qPCR was performed using synthetic primers and SYBR Green (Thermo, Rockford, IL, USA) with an IQ5 Detection System. After incubating at 50 °C for 2 min and 95 °C for 10 min, the samples were subjected to 40 cycles of 95 °C for 15 s and 60 °C for 1 min. GAPDH was used as an endogenous control gene. The sequences of the primers specific for the P2X7R, NLRP3 and GADPH were as follows:

- P2X7R, 5′-CTACTCTTCGGTGGGGGCTT-3′ (forward primer),
- P2X7R, 5′-CTCTGGATCCGGGTGACTTT-3′ (reverse primer);
- NLRP3, 5′-CTGCATGCCGTATCTGGTTG-3′ (forward primer),
- NLRP3, 5′-GCTGAGCAAGCTAAAGGCTTC-3′ (reverse primer);
- GAPDH, 5′-AGACAGCCGCATCTTCTTGT-3′ (forward primer),
- GAPDH, 5′- TGATGGCAACAATGTCCACT-3′ (reverse primer).

### Western blot analysis

Samples, including perihaematomal brain tissues, cell lysates and medium, were collected and subjected to western blot analysis, as described in our previous studies [16, 17]. The following primary antibodies were used: rabbit polyclonal anti-CBS antibody (1:200, Santa Cruz Biotechnology, Santa Cruz, CA, USA), rabbit polyclonal anti-P2X7R antibody (1:1000, Alomone Labs, Jerusalem, Israel), rabbit polyclonal anti-NLRP3 antibody (1:200, Santa Cruz Biotechnology, Santa Cruz, CA, USA), rabbit polyclonal anti-ASC antibody (1:500, Abcam, Cambridge, MA, USA), mouse monoclonal anti-caspase-1 p20 antibody (1:200, Santa Cruz Biotechnology, CA, USA), rabbit polyclonal anti-IL-1β antibody (1:1000, Millipore, Billerica, MA, USA), rabbit polyclonal anti-myeloperoxidase antibody (anti-MPO, 1:500, Abcam, Cambridge, MA, USA) and goat polyclonal anti-Iba1 antibody (1:600, Abcam, Cambridge, MA, USA). GAPDH (1:1000, Cell Signalling Technology, Danvers, MA, USA) was used as a loading control. The proteins were detected on nitrocellulose membranes with enhanced chemiluminescence reagents (GE Healthcare, Beijing, China), and the blot bands were quantified by densitometry with Image J software (National Institutes of Health, Bethesda, MD, USA). The results are expressed as a relative density ratio, which was normalized to the mean value of the vehicle or control group.

### Immunofluorescence staining

Frozen sections (20-μm thickness) were obtained from each group at 1 day after ICH, for immunofluorescence staining as described previously [17], and washed with a 0.01 M phosphate buffer solution after 30 min of heating at 37 °C. The sections were incubated for 30 min in 5% bovine serum albumin and then incubated at 4 °C overnight with the following primary antibodies: rabbit polyclonal anti-CBS antibody (1:100, Santa Cruz Biotechnology, Santa Cruz, CA, USA), rabbit polyclonal anti-P2X7R antibody (1:500, Alomone Labs, Jerusalem, Israel), goat polyclonal anti-Iba1 antibody (1:300, Abcam, Cambridge, MA, USA), and rabbit polyclonal anti-MPO antibody (1:50, Abcam, Cambridge, MA, USA).

The microglial cultures were fixed for 30 min in 4% paraformaldehyde. The cells were blocked with 1% bovine serum for 60 min and then incubated overnight at 4 °C with the following primary antibodies: goat polyclonal anti-Iba1 antibody (1:300, Abcam, Cambridge, MA, USA) and rabbit polyclonal anti-P2X7R antibody (1:500, Alomone Labs, Jerusalem, Israel). For the double-staining experiments, the cells were incubated with the above primary antibodies overnight at 4 °C before undergoing a separate incubation with Alexa 488 and Alexa 568 secondary fluorescent antibodies (1:400, Invitrogen, Carlsbad, CA, USA) for 60 min at 37 °C. The nuclei were stained with 4′,6-diamidino-2-phenylindole (DAPI, Thermo Fisher Scientific, Waltham, MA, USA) for 10 min. The sections and cells were observed by microscopy (Zeiss, AxioCam, Germany) or confocal microscopy (LSM780, Zeiss, Jena, Germany).

### TUNEL staining

TUNEL staining was performed to evaluate the cell apoptosis in perihaematomal brain tissues. At 1 day after ICH, TUNEL staining was performed with an in situ apoptosis detection kit (Roche Molecular Biochemicals, Mannheim, Germany), according to the manufacturer’s instruction. Briefly, selected sections were pretreated with 20 mg/mL proteinase-K in 10 mM Tris-HCl at 37 °C for 15 min and then incubated in 0.3% hydrogen peroxide dissolved in anhydrous methanol for 10 min after being rinsed in phosphate-buffered saline. The sections were then incubated in 0.1% sodium citrate and 0.1% Triton X-100 solution for 2 min at 4 °C. After several washes with phosphate-buffered saline, the sections were incubated with 50 μL of TUNEL reaction mixture with terminal deoxynucleotidyl transferase (TdT) for 60 min at 37 °C under humidified conditions, and the neuronal nuclei were stained with DAPI. Then, the sections were visualized by a confocal microscope. Negative controls were prepared by omitting the TdT enzyme. To evaluate the extent of cell injury, we examined and photographed six microscopic fields in parallel for TUNEL-positive cell counting.

### Statistical analysis

All data are expressed as the mean ± SEM. SPSS 11.5 (SPSS Inc., Chicago, IL, USA) was used for statistical analysis. The Mann–Whitney *U* test was used to compare behaviour and activity scores among the groups. Other data were analysed by one-way ANOVA followed by the Scheffé F test for post hoc analysis. *p* < 0.05 was considered statistically significant.

## Results

### Endogenous H_2_S production after ICH

**Fig. 1:**
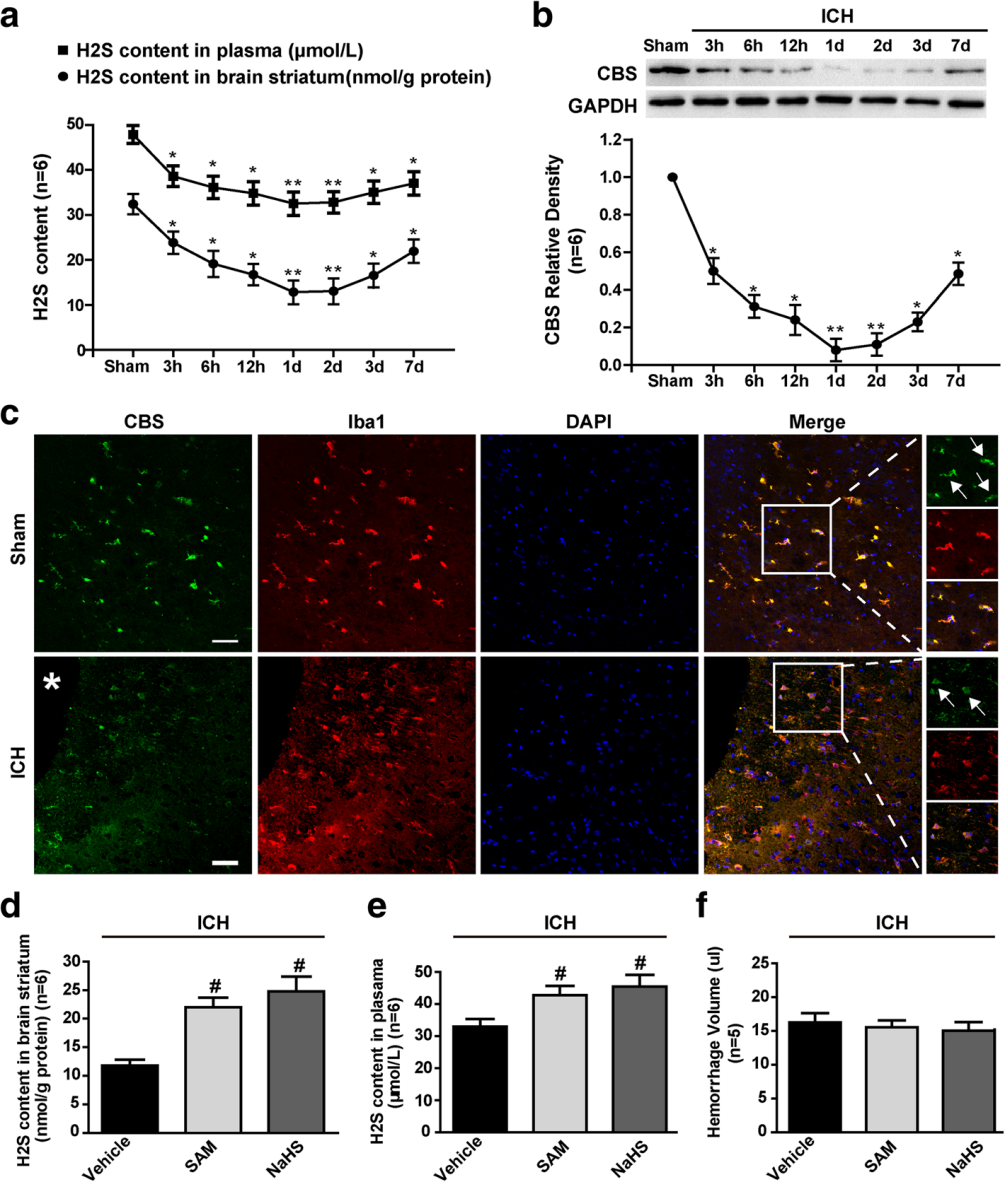
Time course of H_2_S content and CBS expression after ICH in rats. **a** Time course of H_2_S content in the brain striatum and blood plasma at different time points after ICH. H_2_S concentrations in the brain striatum and blood plasma were significantly decreased in ICH-treated rats compared with those in sham-operated rats at all post-ICH time points (*p* < 0.05 vs. sham) and reached a nadir at 1 and 2 days after ICH, respectively (*p* < 0.01 vs. sham). **b** Representative bands and quantitative analysis of the time course of CBS in perihaematomal brain tissues after ICH. CBS, a key enzyme involved in endogenous H_2_S synthesis, exhibited the same trend in its protein levels as H_2_S concentrations in the ipsilateral/right hemisphere after ICH. The relative densities of each protein have been normalized against those of the sham group. **c** Representative photographs of immunofluorescence staining for CBS (green) expression in microglia (Iba-1, red) in sham and the perihaematomal area, as the arrow indicates, at 1 day after ICH. CBS was colocalized with perihaematoma microglia in the ipsilateral striatum, as demonstrated by the presence of Iba1. **d**, **e** H_2_S content in the brain striatum and plasma at 1 day after ICH in the vehicle, SAM and NaHS groups. Both SAM administration and NaHS administration significantly increased H_2_S content in brain striatum (*p* < 0.05 vs. vehicle) and blood plasma (*p* < 0.05 vs. vehicle) at 1 day after ICH. These data also demonstrated that SAM and NaHS administration significantly increased endogenous and exogenous H2S content, respectively, at 1 day after ICH. **f** Quantitative analyses of haemorrhage volume in the vehicle, SAM and NaHS groups at 1 day after ICH. There was no significant difference in haemorrhage volume among the vehicle group, SAM group and NaHS group (*p* > 0.05 vs. vehicle). For haemorrhage volume, *n* = 5 rats per group; for others, *n* = 6 rats per group and time point. Scale bar = 12.5 μm. Data are presented as the mean ± SEM. *p < 0.05 vs. sham, **p < 0.01 vs. sham, #*p* < 0.05 vs. vehicle. CBS cystathionine-β-synthase, DAPI 4′,6-diamidino-2-phenylindole, GAPDH glyceraldehyde 3-phosphate dehydrogenase, ICH intracerebral haemorrhage, SAM *S*-adenosyl-l-methionine

To assess the changes in H_2_S production after ICH, we measured H_2_S concentrations in the brain striatum and blood plasma at 3, 6 and 12 h and 1, 2, 3 and 7 days after ICH (Fig. 1a). We found that H_2_S concentrations in the brain striatum and blood plasma were significantly decreased in ICH-treated rats compared with sham-operated rats at all post-ICH time points (*p* < 0.05 vs. sham) and reached a nadir at 1 and 2 days after ICH, respectively (*p* < 0.01 vs. sham). The western blotting results indicated that CBS, a key enzyme involved in endogenous H_2_S synthesis, exhibited the same trend in its protein levels as H_2_S concentrations in the ipsilateral/right hemisphere after ICH (Fig. 1b). Further investigation with double-immunofluorescence staining revealed that CBS was colocalized with perihaematoma microglia in the ipsilateral striatum, as demonstrated by the presence of Iba1 (Fig. 1c). These data demonstrated that microglia produce endogenous H_2_S after ICH and that ICH significantly inhibits CBS activity.

### Artificial regulation of H_2_S after ICH

Since H_2_S production decreased significantly after ICH, we employed an agonist of CBS, SAM or the classical exogenous H_2_S donor, NaHS, to assess the fundamental functions of H_2_S. Our data indicated that both SAM administration and NaHS administration significantly increased H_2_S content in the brain striatum (*p* < 0.05 vs. vehicle, Fig. 1d) and blood plasma (*p* < 0.05 vs. vehicle, Fig. 1e) at 1 day after ICH. These data also demonstrated that SAM and NaHS administration significantly increased endogenous and exogenous H2S content, respectively, at 1 day after ICH.

There was no significant difference in haemorrhage volume among the vehicle group, SAM group and NaHS group (*p* > 0.05 vs. vehicle, Fig. 1f), demonstrating that our ICH rat models were reproducible and consistent and that the artificial interventions used in this study had no influence on haematoma formation or absorption.

### H_2_S attenuated neurological deficits after ICH

**Fig. 2:**
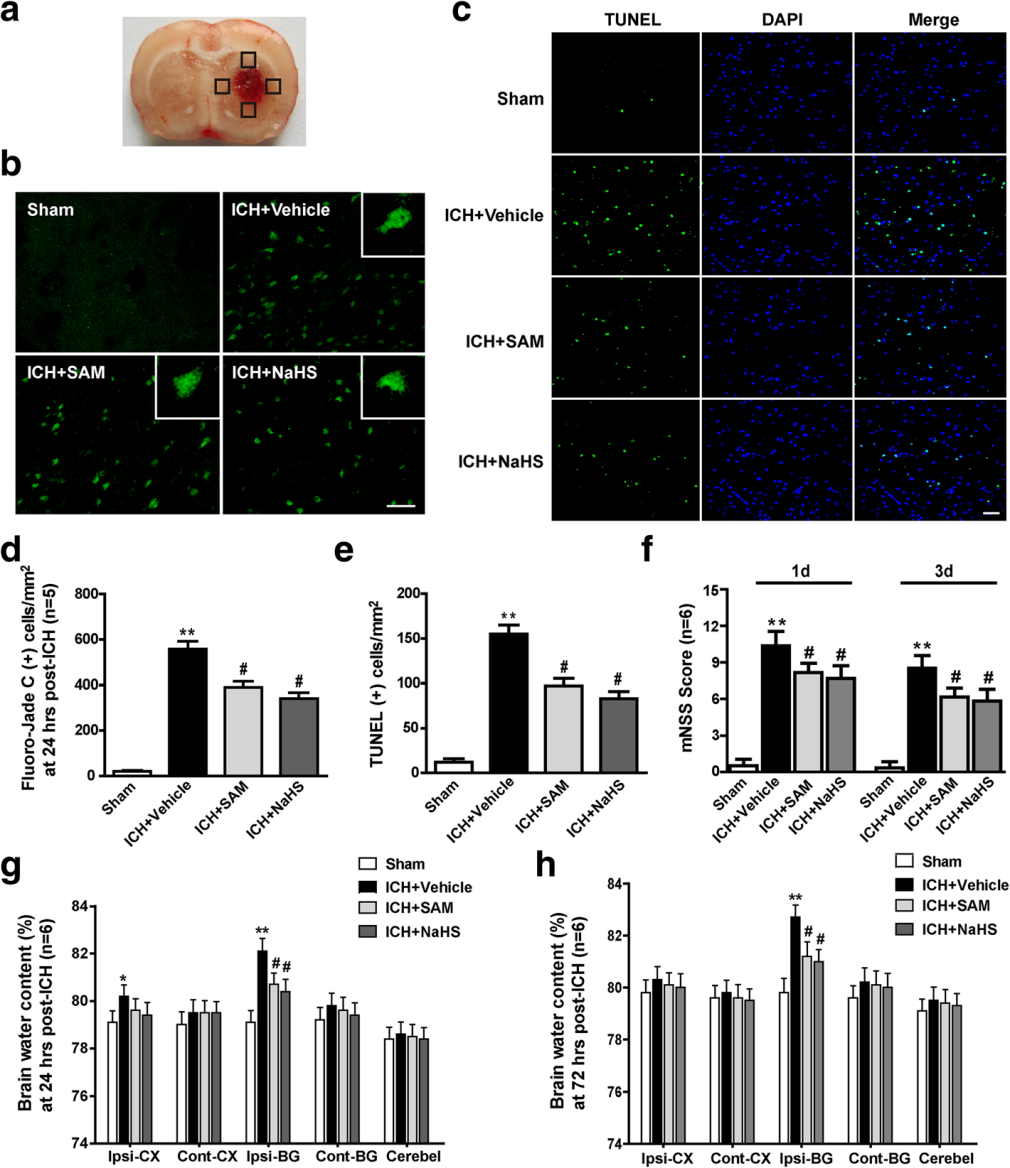
The effects of SAM and NaHS administration on neurological deficits and brain edema at 1 and 3 days after ICH. **a** The schematic diagram shows the four areas (black squares) for Fluoro-Jade C- and TUNEL-positive cell counting in the perihaematomal region. **b** Representative Fluoro-Jade C staining images and quantitative analyses of Fluoro-Jade C-positive neurons surrounding the haematoma in the sham, vehicle, SAM and NaHS groups at 1 day after ICH. **c** Representative images of TUNEL-stained (green) and DAPI-stained (blue) brain sections in the perihaematomal area in the sham, vehicle, SAM and NaHS groups at 1 day following operation. **d**, **e** Quantitative analyses of Fluoro-Jade C-positive neurons and TUNEL-positive cells surrounding the haematoma in the sham, vehicle, SAM and NaHS groups at 1 day after ICH. Fluoro-Jade C staining demonstrated significantly reduced perihaematomal neuronal cell injury as a result of SAM or NaHS administration at 1 day after ICH (*p* < 0.05 vs. vehicle). Compared with the sham group, the vehicle group exhibited an increased number of TUNEL-positive cells in the perihaematomal area (*p* < 0.01). Remarkably, both SAM treatment and NaHS treatment induced a significant reduction in the number TUNEL-positive cells in the perihaematomal area (*p* < 0.05 vs. vehicle). **f** Modified Neurological Severity Scores in the sham, vehicle, SAM and NaHS groups at 1 and 3 days after ICH. SAM and NaHS administration significantly revered the neurological deficits assessed at 1 and 3 days after ICH (*p* < 0.01 vs. sham) at both time points (*p* < 0.05 vs. vehicle). **g**, **h** Brain water content assessment in the sham, vehicle, SAM and NaHS groups at 1 and 3 days after operation. At 1 day after ICH, brain water content was significantly increased in the ipsilateral basal ganglia (*p* < 0.01) and ipsilateral cortex (*p* < 0.05) of the ICH-treated groups compared with that in the sham group. SAM or NaHS treatment significantly reduced brain water content in the ipsilateral basal ganglia (*p* < 0.05 vs. vehicle), but not in the contralateral basal ganglia and ipsilateral cortex (*p* > 0.05 vs. vehicle). Moreover, there were no significant differences in brain water content in the contralateral basal ganglia and ipsilateral cortex in the ICH-treated groups compared to the vehicle group (*p* > 0.05). At 3 days after ICH, brain water content was increased only in the ipsilateral basal ganglia after ICH (*p* < 0.01 vs. sham) and was significantly reduced by both SAM administration and NaHS administration (*p* < 0.05 vs. vehicle). *N* = 6 for mNSS and brain water content assessment; *n* = 5 for Fluoro-Jade C staining; *n* = 4 for TUNEL staining. Scale bar = 50 μm. Data are presented as the mean ± SEM. *p < 0.05 vs. sham; **p < 0.01 vs. sham; #*p* < 0.05 vs. vehicle. SAM *S*-adenosyl-l-methionine, mNSS Modified Neurological Severity Score, ICH intracerebral haemorrhage, TUNEL terminal deoxynucleotidyl transferase dUTP nick end-labelling, DAPI 4′,6-diamidino-2-phenylindole, Ipsi-CX ipsilateral cortex, Cont-CX contralateral cortex, Ipsi-BG ipsilateral basal ganglia, Cont-BG contralateral basal ganglia, Cerebel cerebellum

Fluoro-Jade C staining demonstrated significantly reduced perihaematomal neuronal cell injury as a result of SAM or NaHS administration at 1 day after ICH (*p* < 0.05 vs. vehicle, Fig. 2a, b, d). In addition, compared with the sham group, the vehicle group exhibited an increased number of TUNEL-positive cells in the perihaematomal area (*p* < 0.01, Fig. 2a, c, e). Remarkably, both SAM treatment and NaHS treatment induced a significant reduction in the number of TUNEL-positive cells in the perihaematomal area (*p* < 0.05 vs. vehicle, Fig. 2a, c, e). SAM and NaHS administration significantly revered the neurological deficits assessed at 1 and 3 days after ICH (*p* < 0.01 vs. sham, Fig. 2f) at both time points (*p* < 0.05 vs. vehicle, Fig. 2f).

At 1 day after ICH, brain water content was significantly increased in the ipsilateral basal ganglia (*p* < 0.01) and ipsilateral cortex (*p* < 0.05) of the ICH-treated groups compared with the sham group (Fig. 2g). SAM or NaHS treatment significantly reduced brain water content in the ipsilateral basal ganglia (*p* < 0.05 vs. vehicle, Fig. 2g), but not in the contralateral basal ganglia and ipsilateral cortex (*p* > 0.05 vs. vehicle, Fig. 2g). Moreover, there were no significant differences in brain water content in the contralateral basal ganglia and ipsilateral cortex in the ICH-treated groups compared to the vehicle group (*p* > 0.05, Fig. 2g). At 3 days after ICH, brain water content was increased only in the ipsilateral basal ganglia after ICH (*p* < 0.01 vs. sham, Fig. 2h) and was significantly reduced by both SAM administration and NaHS administration (*p* < 0.05 vs. vehicle, Fig. 2h).

### H_2_S inhibited ICH-induced microglia accumulation, NLRP3 inflammasome activation and subsequent IL-1β release

**Fig. 3:**
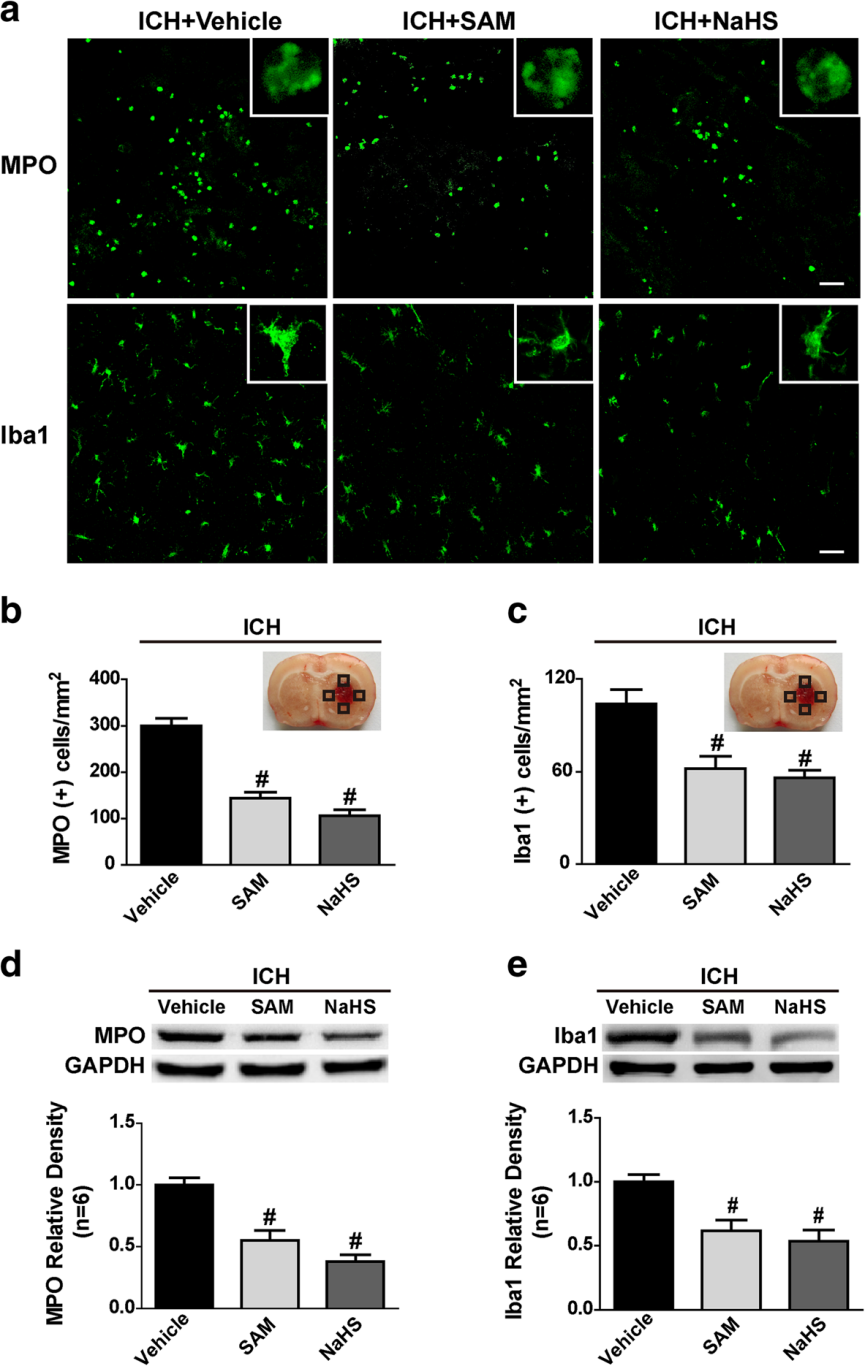
The effects of SAM and NaHS administration on neutrophil infiltration and microglia accumulation at 1 day after ICH. **a** Representative photographs of immunofluorescence staining of MPO immuno-labelled neutrophils (green) and Iba1 immuno-labelled microglia (green) in the perihaematomal area in the vehicle, SAM and NaHS groups at 1 day after ICH. **b**, **c** Quantitative analysis of MPO and Iba1-positive cells in the perihaematomal region in the vehicle, SAM and NaHS groups at 1 day after ICH. SAM and NaHS treatment significantly reduced the number of MPO-positive and Iba1-positive cells in the perihaematomal area in the ICH-treated groups compared to the vehicle group. **d**, **e** Representative bands and quantitative analysis of MPO and Iba1 protein levels in the perihaematomal tissues in the vehicle, SAM and NaHS groups at 1 day after ICH. MPO and Iba1 protein levels in the ipsilateral hemisphere were markedly reduced after SAM and NaHS treatment in the ICH-treated groups compared to the vehicle group. The relative densities of each protein have been normalized against those of the vehicle group. For western blotting, *n* = 6 rats per group; for others, *n* = 4 rats per group. Scale bar = 25 μm. Data are presented as the mean ± SEM. #*p* < 0.05 vs. vehicle. MPO myeloperoxidase, SAM *S*-adenosyl-l-methionine, ICH intracerebral haemorrhage

Since the key enzyme for the endogenous synthesis of H2S, CBS, colocalized with microglia, as illustrated above, we investigated the effects of SAM and NaHS administration on neutrophil infiltration and microglia accumulation. We detected myeloperoxidase (MPO) and Iba1 levels in brain tissue by immunostaining, as well as western blotting, 1 day following ICH. Immunostaining (Fig. 3a) indicated that SAM and NaHS treatment significantly reduced the number of MPO-positive and Iba1-positive cells in the perihaematomal area in the ICH-treated groups compared to the vehicle group (*p* < 0.05, Fig. 3b, c). Consistently, the western blotting results showed that MPO and Iba1 protein levels in the ipsilateral hemisphere were markedly reduced after SAM and NaHS treatment in the ICH-treated groups compared to the vehicle group (*p* < 0.05, Fig. 3d, e).

**Fig. 4:**
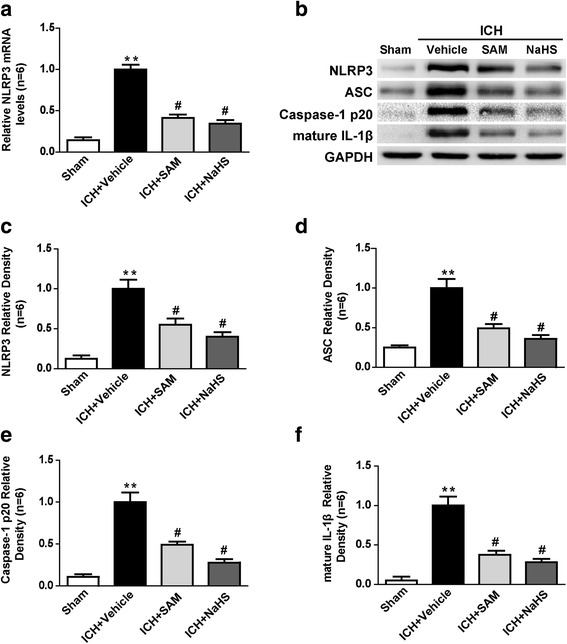
The effects of SAM and NaHS administration on NLRP3 inflammasome activation and IL-1β maturation. **a** Quantitative analysis of NLRP3 mRNA levels by qPCR in the sham, vehicle, SAM and NaHS groups at 1 day after ICH. NLRP3 mRNA levels were apparently increased in the vehicle group at 1 day after ICH (*p* < 0.01 vs. sham). SAM and NaHS treatment significantly reduced NLRP3 mRNA expression in their respective treatment groups compared to the vehicle group (*p* < 0.05). The relative densities of each mRNA have been normalized against those of the vehicle group. **b**–**f** Representative bands and quantitative analysis of NLRP3, ASC, caspase-1 p20 subunit and mature IL-1β levels in the perihaematomal tissues of the sham, vehicle, SAM and NaHS groups at 1 day after ICH. The protein levels of the NLRP3 inflammasome components, including the protein levels of NLRP3, ASC and caspase-1, and the levels of IL-1β production were evidently elevated in the vehicle group at 1 day after ICH (*p* < 0.01 vs. sham). SAM and NaHS treatment significantly suppressed NLRP3 inflammasome component expression and the subsequent secretion of mature IL-1β (*p* < 0.05 vs. vehicle). The relative densities of each protein have been normalized against those of the vehicle group. *N* = 6 rats per group. Data are presented as the mean ± SEM. **p < 0.01 vs. sham; #*p* < 0.05 vs. vehicle. SAM *S*-adenosyl-l-methionine, GAPDH glyceraldehyde 3-phosphate dehydrogenase, ICH intracerebral haemorrhage, IL interleukin, NLRP3 pyrin domain-containing 3, ASC adaptor protein apoptosis-associated speck-like protein containing a CARD

To investigate the effects of SAM and NaHS on NLRP3 inflammasome activation, we first detected NLRP3 messenger RNA (mRNA) levels by qPCR. NLRP3 mRNA levels were apparently increased in the vehicle group at 1 day after ICH (*p* < 0.01 vs. sham, Fig. 4a). SAM and NaHS treatment significantly reduced NLRP3 mRNA expression in their respective treatment groups compared to the vehicle group (*p* < 0.05, Fig. 4a). Then, we detected the protein levels of the NLRP3 inflammasome components and mature IL-1β by western blotting. The protein levels of the NLRP3 inflammasome components, including the protein levels of NLRP3, ASC and caspase-1, and the levels of IL-1β production were evidently elevated in the vehicle group at 1 day after ICH (*p* < 0.01 vs. sham, Fig. 4b–f). SAM and NaHS treatment significantly suppressed NLRP3 inflammasome component expression and the subsequent secretion of mature IL-1β (*p* < 0.05 vs. vehicle, Fig. 4b–f).

### H_2_S suppressed P2X7R expression on microglia after ICH

**Fig. 5:**
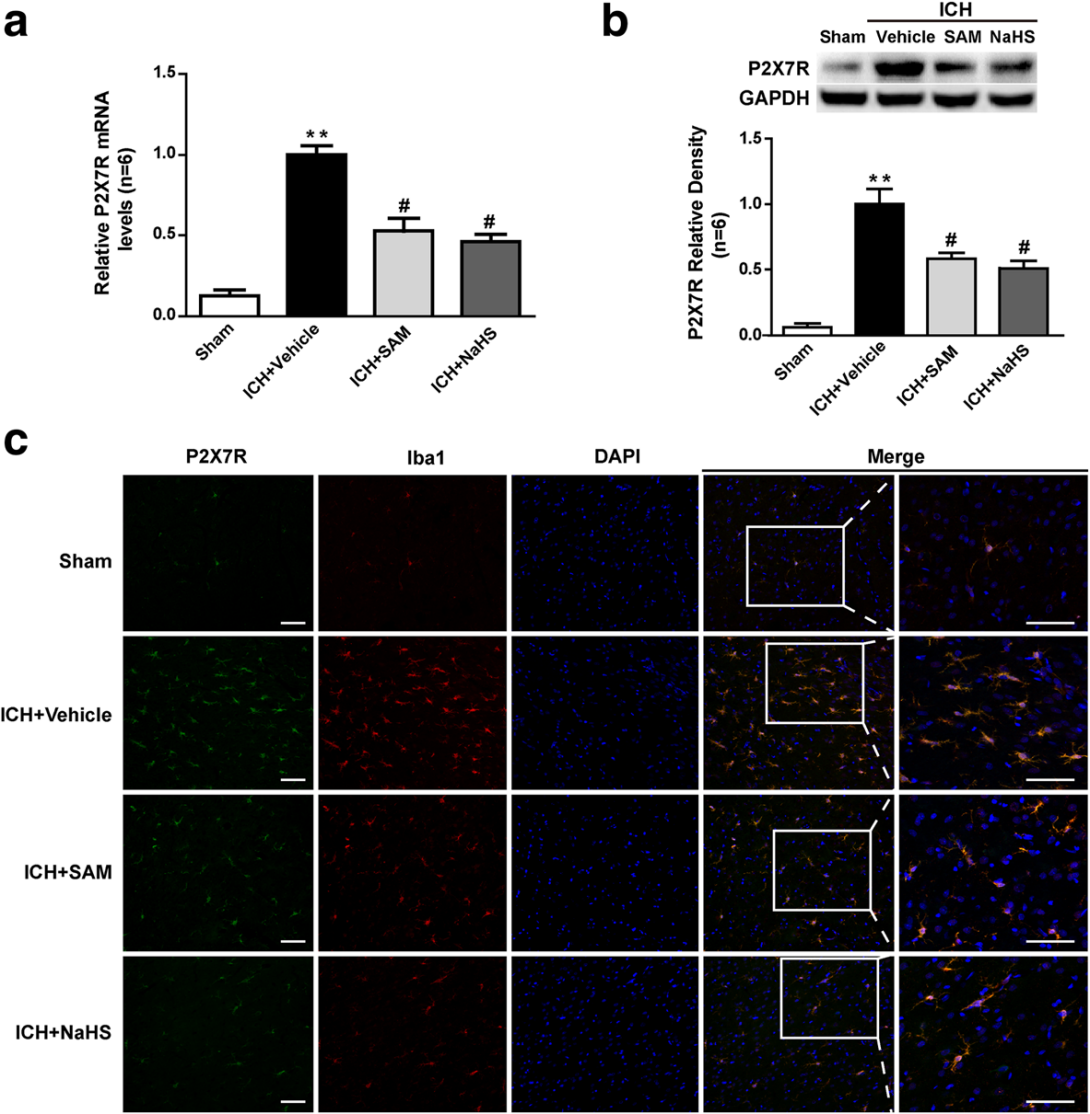
The effects of SAM and NaHS administration on P2X7R expression at 1 day after ICH. **a** Quantitative analysis of P2X7R mRNA levels by qPCR in the sham, vehicle, SAM and NaHS groups at 1 day after ICH. The qPCR results revealed that ICH-induced P2X7R mRNA expression was evidently elevated in the periphery of the haemorrhage at 1 day after injury (*p* < 0.01 vs. sham). Both SAM treatment and NaHS treatment significantly reduced P2X7R mRNA expression in their respective treatment groups compared to the vehicle group (*p* < 0.05). The relative densities of each mRNA have been normalized against those of the vehicle group. **b** Representative bands and quantitative analysis of P2X7R expression in the perihaematomal tissues in the sham, vehicle, SAM and NaHS groups at 1 day after ICH. P2X7R protein expression was upregulated at 1 day after ICH induction (*p* < 0.01 vs. sham) and SAM and NaHS administration reversed this trend, as demonstrated by western blot analysis (*p* < 0.05 vs. vehicle). The relative densities of each protein have been normalized against those of the vehicle group. **c** Representative photographs of Iba1-positive cells co-labelled with P2X7R in the perihaematomal tissues of the sham, vehicle, SAM and NaHS groups at 1 day after ICH. Few P2X7R-positive microglia (Iba1-positive cells) were detected in the sham group. However, many more activated P2X7R-positive microglia were observed in the ICH-treated groups post-ICH. SAM and NaHS administration inhibited the increases in the numbers of P2X7R-positive microglia that occurred after ICH induction. *N* = 6 rats per group, scale bar = 40 μm. Data are the mean ± SEM. **p < 0.01 vs. sham, #*p* < 0.05 vs. vehicle. SAM *S*-adenosyl-l-methionine, GAPDH glyceraldehyde 3-phosphate dehydrogenase, ICH intracerebral haemorrhage, DAPI 4′,6-diamidino-2-phenylindole

The qPCR results revealed that ICH-induced P2X7R mRNA expression was evidently elevated in the periphery of the haemorrhage at 1 day after injury (*p* < 0.01 vs. sham, Fig. 5a). Both SAM treatment and NaHS treatment significantly reduced P2X7R mRNA expression in their respective treatment groups compared to the vehicle group (*p* < 0.05, Fig. 5a). Likewise, P2X7R protein expression was upregulated at 1 day after ICH induction (*p* < 0.01 vs. sham, Fig. 5b), and SAM and NaHS administration reversed this trend, as demonstrated by western blot analysis (*p* < 0.05 vs. vehicle, Fig. 5b).

Immunofluorescent characterization of the brain sections at 1 day after ICH and subsequent confocal analyses were performed to evaluate P2X7R and microglia cell colocalization. Few P2X7R-positive microglia (Iba1-positive cells) were detected in the sham group. However, many more activated P2X7R-positive microglia were observed in the ICH-treated groups post-ICH. SAM and NaHS administration inhibited the increases in the numbers of P2X7R-positive microglia that occurred after ICH induction (Fig. 5c).

**Fig. 6:**
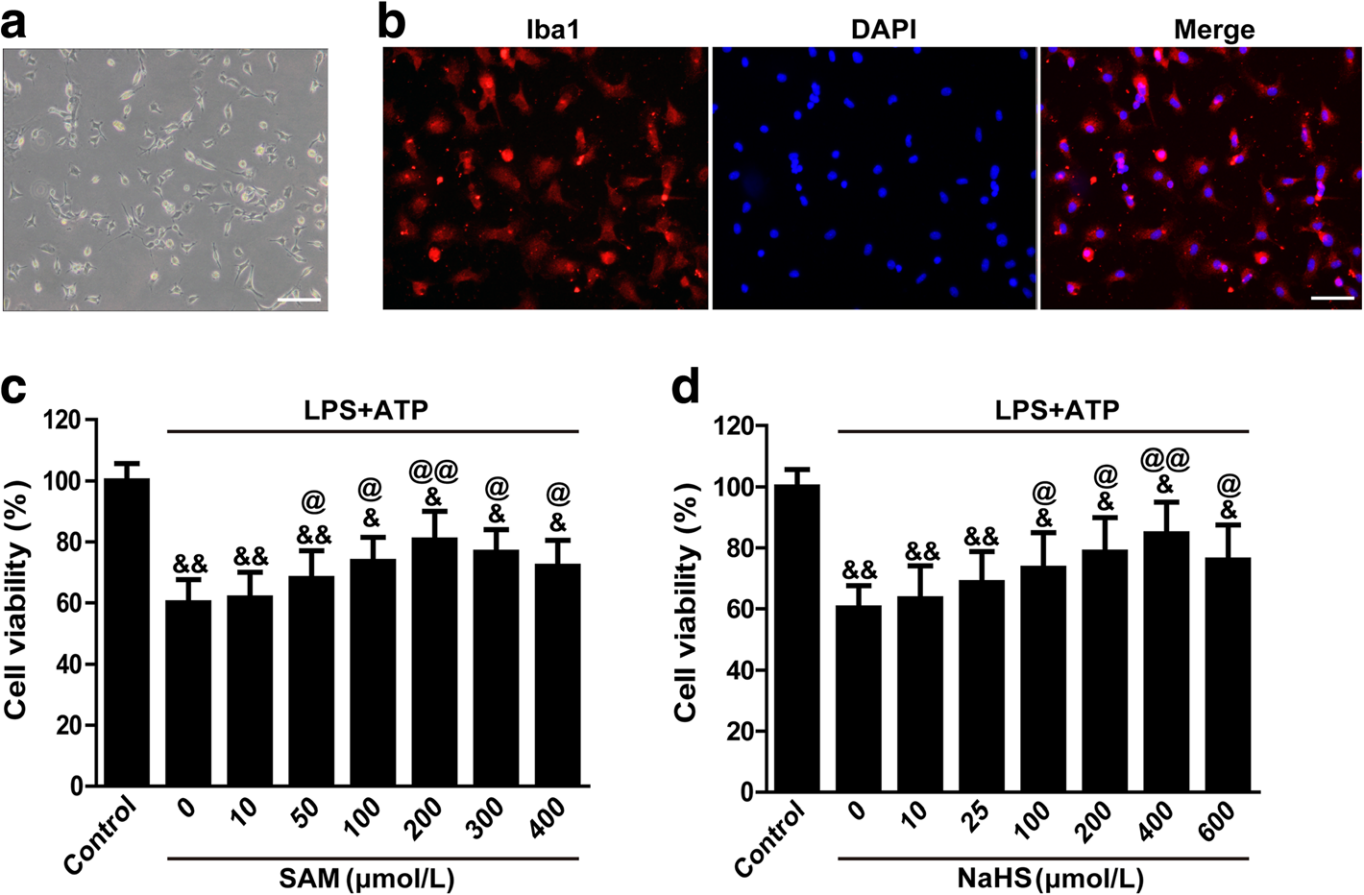
Primary cultured rat microglial cells and the effects of SAM and NaHS on cell viability following LPS and ATP stimulation. **a** Microscope-captured image of microglial cells and **b** representative photographs of immunofluorescence staining for Iba-1-positive primary cultured rat microglia. **c**, **d** The effects of different concentrations of SAM and NaHS on the viability of rat primary cultured microglial cells. LPS and ATP stimulation substantially inhibited cell viability, as demonstrated by CCK-8 assay (*p* < 0.01 vs. control). These cytotoxic effects were attenuated in a concentration-dependent manner by SAM at concentrations ranging from 50 to 400 μM, and the maximum response was achieved at a concentration of 200 μM (*p* < 0.01 vs. control). Similar effects were observed with NaHS administration. NaHS exerted its maximal effect at a concentration of 400 μM (*p* < 0.01 vs. control). *N* = 3 per group, scale bars = 75 μm. Data are presented as the mean ± SEM. &&*p* < 0.01 vs. control; &*p* < 0.05 vs. control; @@*p* < 0.01 vs. LPS + ATP (with 0 μmol SAM); @*p* < 0.05 vs. LPS + ATP (with 0 μmol SAM). SAM *S*-adenosyl-l-methionine, DAPI 4′,6-diamidino-2-phenylindole

To verify the involvement of the P2X7R in the suppression of NLRP3 inflammasome activation by H2S in microglia, we cultured primary microglial cells under in vitro conditions characterized by artificial inflammation and P2X7R activation facilitated by combining LPS and ATP in the culture mediums. First, to verify the primary cultured rat microglial cells, we seeded a subset of cells onto glass slides for characterization by immunofluorescence, which showed that the primary microglial cells were Iba1-positive cells (Fig. 6a, b). LPS and ATP stimulation substantially inhibited cell viability, as demonstrated by the CCK-8 assay (*p* < 0.01 vs. control, Fig. 6c). These cytotoxic effects were attenuated in a concentration-dependent manner by SAM at concentrations ranging from 50 to 400 μM, and the maximum response was achieved at a concentration of 200 μM (*p* < 0.01 vs. control, Fig. 6c). Similar effects were observed with NaHS administration. NaHS exerted its maximal effect at a concentration of 400 μM (*p* < 0.01 vs. control, Fig. 6d). These data suggested that H_2_S protected the microglial cells against LPS and ATP stimulation-induced cell injury, results consistent with those of our in vivo study, especially the results of our TUNEL staining assay. SAM (at a concentration of 200 μM) or NaHS (at a concentration of 400 μM) was added to produce endogenous and exogenous H2S for the following in vitro study.

**Fig. 7:**
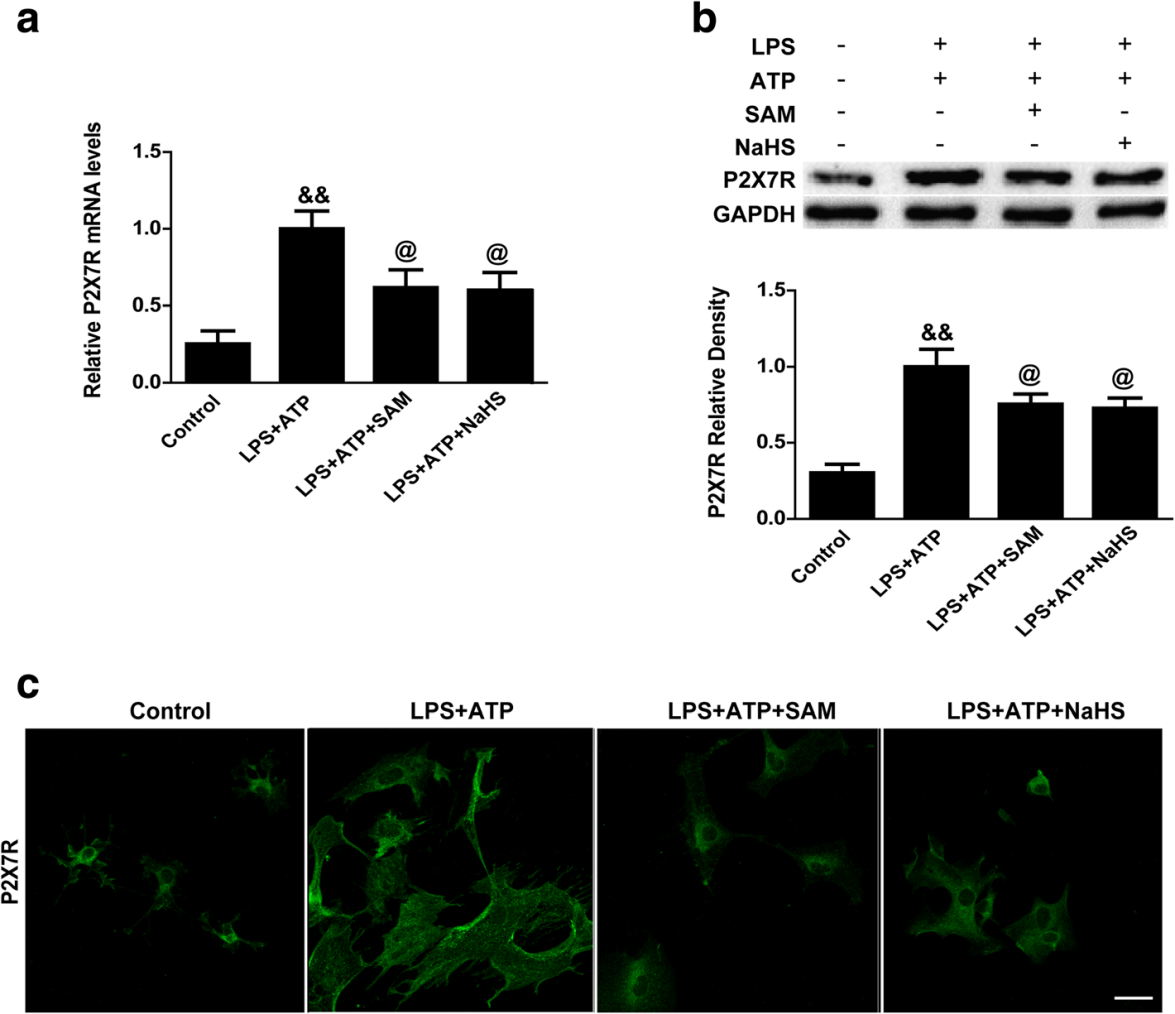
The effects of SAM and NaHS administration on P2X7R expression in primary microglial cells. **a** Quantitative analysis of P2X7R mRNA levels by qPCR in the control, LPS + ATP, LPS + ATP + SAM and LPS + ATP + NaHS groups. P2X7R mRNAs were evidently elevated after LPS and ATP stimulation (*p* < 0.01 vs. control) and that both SAM treatment and NaHS treatment significantly suppressed these upregulations (*p* < 0.05 (**a**, **b**)). The relative densities of each mRNA have been normalized against those of the LPS + ATP group. **b** Representative bands and quantitative analysis of P2X7R in the control, LPS + ATP, LPS + ATP + SAM and LPS + ATP + NaHS groups. P2X7R protein levels were evidently elevated after LPS and ATP stimulation (*p* < 0.01 vs. control) and that both SAM treatment and NaHS treatment significantly suppressed these upregulations (*p* < 0.05). The relative densities of each protein have been normalized against those of the LPS + ATP group. **c** Representative photographs of P2X7R-positive cells in the control, LPS + ATP, LPS + ATP + SAM and LPS + ATP + NaHS groups. The numbers of P2X7R-positive cells were increased and that the cells were visually identical to activated microglia after LPS and ATP stimulation. Both SAM treatment and NaHS treatment significantly alleviated these changes. *N* = 3 per group, scale bar = 50 μm. Data are the mean ± SEM. **p < 0.01 vs. sham, #*p* < 0.05 vs. vehicle, &&*p* < 0.01 vs. control, @*p* < 0.05 vs. LPS + ATP. SAM *S*-adenosyl-l-methionine, DAPI 4′,6-diamidino-2-phenylindole

Subsequently, qPCR and western blotting were carried out to detect P2X7R mRNA and protein expression. The results of those experiments revealed that both P2X7R mRNA and P2X7R protein levels were evidently elevated after LPS and ATP stimulation (*p* < 0.01 vs. control, Fig. 7a, b) and that both SAM treatment and NaHS treatment significantly suppressed these upregulations (*p* < 0.05, Fig. 7a, b). Immunostaining also indicated that the numbers of P2X7R-positive cells were increased and that the cells were visually identical to activated microglia after LPS and ATP stimulation (Fig. 7c). Both SAM treatment and NaHS treatment significantly alleviated these changes (Fig. 7c).

### H_2_S reversed the detrimental effects of P2X7R overexpression

**Fig. 8:**
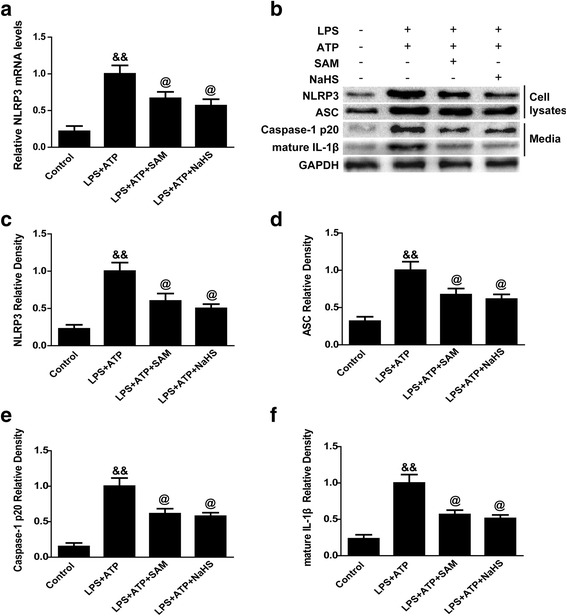
The effects of SAM and NaHS administration on NLRP3 inflammasome activation in primary microglial cells. **a** Quantitative analysis of NLRP3 mRNA levels by qPCR in the control, LPS + ATP, LPS + ATP + SAM and LPS + ATP + NaHS groups. The relative densities of each mRNA have been normalized against those of the LPS + ATP group. LPS and ATP stimulation significantly enhanced NLRP3 mRNA levels (*p* < 0.01 vs. control). Both SAM and NaHS treatment inhibited this enhancement (*p* < 0.05 vs. LPS + ATP). **b**–**f** Representative bands and quantitative analysis of NLRP3 and ASC expression in the microglial cell lysates and caspase-1 p20 subunit and mature IL-1β levels in the medium and in the control, LPS + ATP, LPS + ATP + SAM and LPS + ATP + NaHS groups. Compared with the control group, LPS and ATP stimulation increased the protein levels of the NLRP3 inflammasome components in the LPS + ATP group, leading to increased levels of IL-1β and caspase-1 release in the medium (*p* < 0.01), but SAM and NaHS treatment significantly suppressed these upregulations (*p* < 0.05). The relative densities of each protein have been normalized against those of the LPS + ATP group. *N* = 3 per group, Data are presented as the mean ± SEM. &&*p* < 0.01 vs. control; @*p* < 0.05 vs. LPS + ATP. SAM *S*-adenosyl-l-methionine, GAPDH glyceraldehyde 3-phosphate dehydrogenase, IL interleukin, NLRP3 pyrin domain-containing 3, ASC adaptor protein apoptosis-associated speck-like protein containing a CARD

To study the effects of H2S on microglial NLRP3 inflammasome activation in vitro, we applied qPCR to detect NLRP3 mRNA levels in the microglial cell lysates, and then, we carried out western blotting to detect NLRP3 and ASC protein levels in the microglial cell lysates, as well as caspase-1 p20 and mature IL-1β levels in the medium, under LPS and ATP stimulation. LPS and ATP stimulation significantly enhanced NLRP3 mRNA levels (*p* < 0.01 vs. control, Fig. 8a). Both SAM and NaHS treatment inhibited this enhancement (*p* < 0.05 vs. LPS + ATP, Fig. 8a). Compared with the control group, LPS and ATP stimulation increased the protein levels of the NLRP3 inflammasome components in the LPS + ATP group, leading to increased levels of IL-1β and caspase-1 release in the medium (*p* < 0.01, Fig. 6b–f), but SAM and NaHS treatment significantly suppressed these upregulations (*p* < 0.05, Fig. 8b–f).

**Fig. 9:**
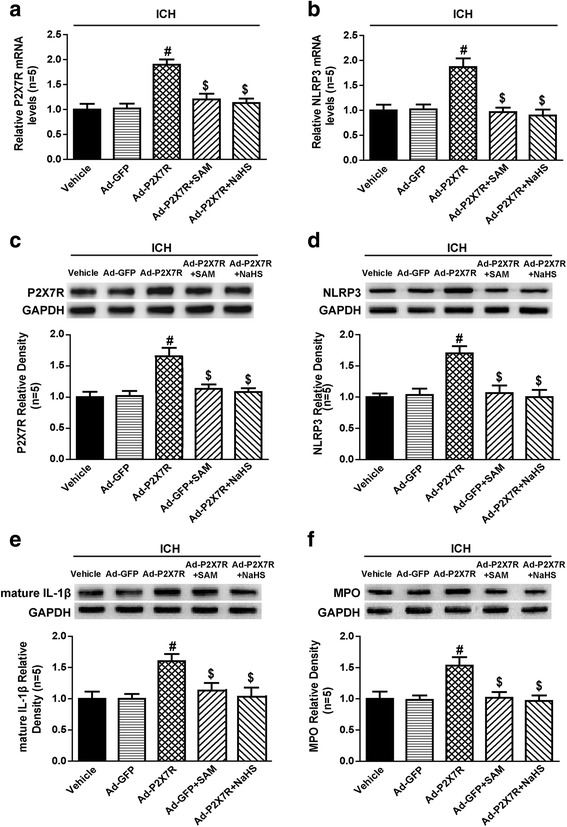
The effects of SAM and NaHS administration on NLRP3 inflammasome activation and MPO expression in P2X7R-overexpressing rats at 1 day after ICH. **a**, **b** Quantitative analysis of P2X7R and NLRP3 mRNA levels by qPCR in the vehicle, Ad-GFP, Ad-P2X7R, Ad-P2X7R + SAM and Ad-P2X7R + NaHS groups at 1 day after ICH. The mRNA levels of the P2X7R and NLRP3 were upregulated in Ad-P2X7R group (*p* < 0.05 vs. vehicle), but not in Ad-GFP group (*p* > 0.05 vs. vehicle), at 1 day after ICH. Administration of both SAM and NaHS inhibited the increase in P2X7R expression (*p* < 0.05 vs. Ad-P2X7R). The relative densities of each mRNA have been normalized against those of the vehicle group. **c**–**e** Representative bands and quantitative analysis of P2X7R, NLRP3 and mature IL-1β levels in the vehicle, Ad-GFP, Ad-P2X7R, Ad-P2X7R + SAM and Ad-P2X7R + NaHS groups at 1 day after ICH. The relative densities of each protein have been normalized against those of the vehicle group. **f** Representative bands and quantitative analysis of MPO levels in the vehicle, Ad-GFP, Ad-P2X7R, Ad-P2X7R + SAM and Ad-P2X7R + NaHS groups at 1 day after ICH. The relative densities of each protein have been normalized against those of the vehicle group. Western blotting performed at 1 day after ICH indicated that similar increases in the protein levels of the P2X7R and NLRP3 were induced by P2X7R overexpression and were suppressed by SAM and NaHS administration. Moreover, the protein levels of mature IL-1β and MPO were significantly elevated by overexpressing P2X7R at 1 day after ICH in the indicated group compared to the vehicle group (*p* < 0.05), but SAM or NaHS administration inhibited these increases (*p* < 0.05 vs. Ad-P2X7R). *N* = 5 rats per group. Data are presented as the mean ± SEM. #*p* < 0.05 vs. vehicle; $*p* < 0.05 vs. Ad-P2X7R. SAM *S*-adenosyl-l-methionine, NLRP3 pyrin domain-containing 3, GAPDH glyceraldehyde 3-phosphate dehydrogenase, ICH intracerebral haemorrhage, IL interleukin, Ad adenovirus, GFP green fluorescent protein, MPO myeloperoxidase

To clarify the role of the P2X7R in the activation of the NLRP3 inflammasome and the subsequent processing of IL-1β, we generated recombinant adenoviruses to overexpress the P2X7R in vivo. The qPCR results revealed that the mRNA levels of the P2X7R and NLRP3 were upregulated in the Ad-P2X7R group (*p* < 0.05 vs. vehicle, Fig. 9a, b), but not in the Ad-GFP group (*p* > 0.05 vs. vehicle, Fig. 9a, b), at 1 day after ICH. Administration of both SAM and NaHS inhibited the increase in P2X7R expression (*p* < 0.05 vs. Ad-P2X7R, Fig. 9a, b). Western blotting performed at 1 day after ICH indicated that similar increases in the protein levels of the P2X7R and NLRP3 were induced by P2X7R overexpression and were suppressed by SAM and NaHS administration (Fig. 9c, d). Moreover, the protein levels of mature IL-1β and MPO were significantly elevated by overexpressing P2X7R at 1 day after ICH in the indicated group compared to the vehicle group (*p* < 0.05, Fig. 9e, f), but SAM or NaHS administration inhibited these increases (*p* < 0.05 vs. Ad-P2X7R, Fig. 9e, f).

**Fig. 10:**
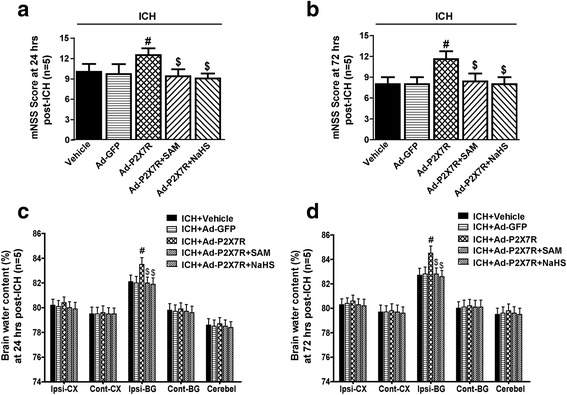
The effects of SAM and NaHS administration on neurological outcomes and brain edema in P2X7R-overexpressing rats at 1 and 3 days after ICH. **a**, **b** Modified Neurological Severity Score in the vehicle, Ad-GFP, Ad-P2X7R, Ad-P2X7R + SAM and Ad-P2X7R + NaHS groups at 1 and 3 days after ICH. **c**, **d** Brain water content assessment in the vehicle, Ad-GFP, Ad-P2X7R, Ad-P2X7R + SAM and Ad-P2X7R + NaHS groups at 1 and 3 days after ICH. Regarding the 3-day study of neurological outcomes and brain oedema in the setting of P2X7R overexpression after ICH, the neurological deficits and brain oedema were worse in the Ad-P2X7R group (*p* < 0.05), but not in the Ad-GFP group (*p* > 0.05 vs. vehicle), than in the vehicle group. Neurological deficits and brain oedema were significantly alleviated in the Ad-P2X7R + SAM and Ad-P2X7R + NaHS groups (*p* < 0.05 vs. Ad-P2X7R). *N* = 5 rats per group. Data are presented as the mean ± SEM. #*p* < 0.05 vs. vehicle; $*p* < 0.05 vs. Ad-P2X7R. SAM *S*-adenosyl-l-methionine, ICH intracerebral haemorrhage, Ad adenovirus, GFP green fluorescent protein

Additionally, regarding the 3-day study of neurological outcomes and brain oedema in the setting of P2X7R overexpression after ICH, we found that neurological deficits (Fig. 10a, b) and brain oedema (Fig. 10c, d) were worse in the Ad-P2X7R group (*p* < 0.05), but not in the Ad-GFP group (*p* > 0.05 vs. vehicle, Fig. 10a–d), than in the vehicle group. Neurological deficits and brain oedema were significantly alleviated in the Ad-P2X7R + SAM and Ad-P2X7R + NaHS groups (*p* < 0.05 vs. Ad-P2X7R, Fig. 10a–d). These results indicated that the P2X7R played a pivotal role in H_2_S-mediated anti-NLRP3 inflammasome activation and neuroprotection after ICH.

## Discussion

In this study, we demonstrated that ICH induced significant downregulation of endogenous H_2_S production in the rat brain. This downregulation may be the result of a decrease in CBS protein levels. Administration of SAM, the CBS-specific agonist, or NaHS, a classical exogenous H_2_S donor, not only restored brain and plasma H_2_S content but also attenuated brain oedema, neutrophil infiltration, microglial accumulation, neurological deficits and neuronal apoptosis at 1 day post-ICH by inhibiting the P2X7R/NLRP3 inflammasome signalling cascade. Endogenous H_2_S production, which was mainly driven by microglial cells and the above treatments, was verified by adenovirus-mediated P2X7 receptor overexpression and an in vitro study involving a primary microglial cell system. To our knowledge, we are the first to report that endogenous H_2_S served as an anti-neuroinflammatory gasotransmitter in microglia to attenuate secondary brain injury mediated by the P2X7R/NLRP3 inflammasome signalling pathway after experimental ICH.

It is well known that the neuroinflammatory response plays an important role in the pathogenesis of ICH and that this response involves cell–cell connections between activated glial cells and neurons in the vascular neural network [21, 22]. Microglia are specialized macrophages residing in the central nervous system. Ample amounts of evidence indicate that they play a crucial role in neuroinflammation. Activated microglia produce and release some pro-inflammatory factors, such as nitric oxide, tumour necrosis factor-α and IL-1β, which aggravate cell death and brain tissue injury further [23, 24]. Previous studies have shown that H_2_S attenuated the inflammatory response in microglia exposed to lipopolysaccharides [25, 26] and demonstrated the ability to attenuate blood-brain barrier permeability and brain oedema after cardiac arrest and resuscitation [27]. However, the origin of endogenous H_2_S has not been fully elucidated. Increasing amounts of attention have been focused on the intestinal microbiome, which produces H_2_S with the ability to cross the blood–brain barrier [28, 29]. In the present study, we found that the predominant H_2_S-generating enzyme, CBS, colocalized with microglia after ICH, which suggested that microglia are sources of endogenous H_2_S in the central neural system. Moreover, the aforementioned reductions in H_2_S content and inhibition of CBS may indicate that the anti-inflammatory effects of H_2_S were impaired after ICH.

The inflammasome is a multiprotein complex that serves as a platform for caspase-1 activation and IL-1β maturation. Our previous study demonstrated that activation of the NLRP3 inflammasome, which contains NLRP3, ASC and caspase-1, makes great contributions to neuroinflammation after ICH [3]. Recently, the NLRP3 inflammasome was reported to be expressed exclusively on microglia and exerts detrimental effects after ICH [4, 6]. Li et al. reported that NaHS administration prevented and partially reversed ozone-induced features of lung inflammation and emphysema by regulating NLRP3 inflammasome activation [30]. Toldo et al. demonstrated that exogenous H_2_S attenuates myocardial ischaemic and inflammatory injury in mice [31]. However, whether H_2_S influences NLRP3 inflammasome activation following ICH remains unclear. In the present study, we employed SAM and NaHS to upregulate endogenous and exogenous H_2_S to examine the effects H_2_S on NLRP3 inflammasome activation both in vivo and in vitro. We demonstrated that both treatments inhibited ICH-induced neutrophil infiltration, microglia accumulation and cell injury, as well as NLRP3 inflammasome activation.

The NLRP3 inflammasome can be activated by many stimuli, including potassium efflux, intracellular calcium level alterations, ubiquitination and reactive oxygen species generation [32]. After ICH, extracellular ATP binds to the P2X7R and triggers potassium efflux, which is a sufficient signal for NLRP3 activation [12, 33]. Recent studies have linked the P2X7R to NLRP3 inflammasome activation in response to diverse inflammatory danger signals [12]. Coincidentally, Gustin et al. demonstrated that the NLRP3 inflammasome is expressed and functional in mouse brain microglia, the same cell in which endogenous H_2_S is produced, but not in astrocytes [34]. Our previous study demonstrated that P2X7R suppression could preserve the blood–brain barrier after ICH [17], and another study found that P2X7R suppression could inhibit ICH-induced NLRP3 inflammasome activation [6]. Therefore, we investigated the involvement of the P2X7R in the effects of H_2_S on NLRP3 inflammasome activation after ICH. The results of this investigation supported our hypothesis.

Moreover, we employed primary microglial cells to identify the specific cell involved in this mechanism. LPS is a classical means of mimicking artificial inflammation in vitro but failed to generate ATP and activate the P2X7R. Thus, we added ATP for the present in vitro studies, which were based on the complications of ICH pathophysiology. We found that LPS + ATP exposure could successfully activate the P2X7R on primary microglia following NLRP3 inflammasome activation, findings consistent with those of the in vitro model of NLRP3 inflammasome activation, which was described previously [35]. Both SAM treatment and NaHS treatment attenuated the increased expression of P2X7R induced by LPS + ATP exposure and alleviated the changes in P2X7R-positive cells exhibiting morphology characteristic of activated microglia, as reported previously [36]. All these data greatly supported the observations of our in vivo study.

Several potential weaknesses of our study should be mentioned. First, apart from facilitating H_2_S formation, SAM exerts a spectrum of biological effects, a number of which have been attributed to its methyl donor properties [37]. For example, a previous study reported that striatal adenosine A2A receptor expression is controlled by SAM-mediated methylation [14]. Therefore, we could not exclude the possibility that other mechanisms contribute to the regulation of P2X7R expression and NLRP3 inflammasome activation by SAM after ICH. Second, H_2_S is known to play an important role in anti-oxidation; thus, it is reasonable to speculate that H_2_S could inactivate the NLRP3 inflammasome by directly clearing reactive oxygen species after ICH, in addition to suppressing P2X7R expression, as was noted in the present study. Finally, although the increased expression of the P2X7R is believed to be the result of accumulation of extracellular ATP from damaged or dying cells after ICH, ATP overflow has not been measured in vivo and in vitro. Further exploration of how H_2_S affects ATP overflow and the subsequent elevations in P2X7R expression is still needed.

## Conclusions

Endogenous H_2_S synthesis, which plays a pivotal role in the P2X7R/NLRP3 inflammasome-associated neuroinflammatory response in the pathogenesis of secondary brain injury, was impaired after ICH. Maintaining appropriate H_2_S concentrations in the central nervous system may represent a potential therapeutic strategy for managing secondary brain injury and its associated neurological deficits after ICH.

## Abbreviations

Ad-GFP: Adenovirus GFP
Ad-P2X7R: Adenovirus P2X7R
ARRIVE: Animal Research: Reporting in Vivo Experiments
ATP: Adenosine triphosphate
CBS: Cystathionine-β-synthase
CCK-8: Cell Counting Kit-8
Cont-BG: Contralateral basal ganglia
Cont-CX: Contralateral cortex
DAPI: 4′,6-Diamidino-2-phenylindole
DMSO: Dimethylsulfoxide
H_2_S: Hydrogen sulphide
HCl: Hydrogen chloride
ICH: Intracerebral haemorrhage
Ipsi-BG: Ipsilateral basal ganglia
Ipsi-CX: Ipsilateral cortex
KMnO_4_: Potassium permanganate
LPS: Lipopolysaccharide
mNSSs: Modified Neurological Severity Scores
NaHS: Sodium hydrosulfide
P2X7R: P2X7 receptor
qPCR: Quantitative polymerase chain reaction
SAM: *S*-adenosyl-l-methionine
SD: Sprague–Dawley
TCA: Trichloroacetic acid
TdT: Terminal deoxynucleotidyl transferase
TUNEL: Terminal deoxynucleotidyl transferase dUTP nick end-labelling
